# PSA Zero Radiographic Disease Progression on PSMA PET/CT

**DOI:** 10.3390/cancers18050831

**Published:** 2026-03-04

**Authors:** Ahmed M. Mahmoud, Carter Day, Eman E. Ahmed, Mohamed E. Ahmed, Rimki Haloi, Mindie Mahon, Yalda Nikanpour, Daniel S. Childs, Jacob J. Orme, Ayse Tuba Kendi, Geoffrey B. Johnson, Eugene D. Kwon, Jack R. Andrews

**Affiliations:** 1 Department of Radiation Oncology, Kansas University Medical Center, Kansas City, KS 66160, USA; 2 Department of Urology, Mayo Clinic, Rochester, MN 55905, USAhaloi.rimki@mayo.edu (R.H.);; 3 Department of Radiology, Division of Nuclear Medicine, Mayo Clinic, Rochester, MN 55905, USA; nikanpour.yalda@mayo.edu (Y.N.);; 4 Department of Medical Oncology, Mayo Clinic, Rochester, MN 55905, USA; 5 Department of Immunology, Mayo Clinic, Rochester, MN 55905, USA; 6 Department of Urology, Mayo Clinic Arizona, Phoenix, AZ 55905, USA; andrews.jack@mayo.edu

**Keywords:** prostate cancer, prostate-specific antigen (PSA), prostate-specific membrane antigen (PSMA), PET/CT

## Abstract

**Background and Objective:** Radiographic progression in prostate cancer (PCa) can occur even when prostate-specific antigen (PSA) levels are undetectable. We aimed to determine the frequency and characteristics of radiographic disease progression (rDP) on PSMA PET/CT in patients with undetectable PSA, referred to as PSA zero rDP. **Methods:** We analyzed the Mayo Clinic PSMA PET Prostate Cancer Registry to identify patients with rDP on PSMA PET/CT despite undetectable PSA levels. Disease progression was confirmed via biopsy or treatment response. The cohort included patients with non-metastatic and metastatic hormone-sensitive disease, as well as those with castration-resistant prostate cancer at the time of imaging. Overall survival (OS) was estimated using the Kaplan–Meier method. Group comparisons were performed with the log-rank test. Univariate Cox regression was used to identify factors associated with poor OS. **Key findings and Limitations:** Among 2141 patients imaged between 2021 and 2023, 257 (12%) had PSA zero rDP. Sixty-one percent had initially localized disease; 39% had de novo metastatic disease. Median (IQR) time from diagnosis to PSA zero rDP was 51.9 (18.4–115.5) months. A total of 184 patients (72%) progressed to castration-resistant PCa. Sites of rDP included bone (57%), visceral (15%), lymph node (18%), and local recurrence (10%). During median follow-up of 8.1 (3.5–11.9) months, 5% of patients died. Only visceral metastases were significantly associated with poorer OS (*p* < 0.0001). **Conclusions and Clinical Implications:** Prostate cancer patients frequently develop metastatic disease with undetectable PSA values. Our findings suggest the use of periodic advanced imaging techniques, irrespective of PSA value, for more prompt detection and early management of disease progress.

## Simple Summary

Some patients with recurrent prostate cancer show disease on imaging even if their PSA tests appear normal. Early detection of recurrence is important to guide treatment and improved outcomes. In our study, we focused on these patients and described their characteristics. PSMA PET/CT scanning was able to detect recurrent prostate cancer in patients with low blood test levels, and the imaging showed disease progression that would not have been apparent from PSA blood tests alone. These findings demonstrated that PSMA PET/CT can identify recurrence even at low PSA levels and provide valuable information for planning treatment and support more precise monitoring and management of prostate cancer.

## 1. Introduction

In the landscape of prostate cancer (PCa) management, the assessment of disease progression has traditionally relied heavily on monitoring serum prostate-specific antigen (PSA) levels, a practice deeply ingrained in clinical practice. PSA serves as a key biomarker for disease activity, guiding treatment decisions and informing prognosis [1,2,3]. However, many reports have shed light on a notable phenomenon in which radiographic disease progression (rDP) occurred in PCa patients with low or undetectable PSA levels, and this finding was reported in both hormone-sensitive prostate cancer (HSPC) and castration-resistant prostate cancer (CRPC) patients [2,4,5]. This divergence between PSA kinetics and radiographic findings challenges the traditional reliance on PSA as the primary indicator of disease status and underscores the intricacies of PCa biology. The recognition of radiographic disease progression in the absence of PSA elevation has significant clinical implications, necessitating a reevaluation of monitoring strategies and treatment paradigms [4]. Historically, achieving a low or undetectable PSA level has been associated with favorable long-term outcomes following various treatments; however, certain tumors may exhibit low PSA secretion and harbor aggressive biological features, resulting in a heterogeneous prognosis among patients with low PSA levels [6,7,8,9,10].

This discordance between PSA kinetics and radiographic findings presents a significant challenge to the prevailing paradigm of PSA-based surveillance. It challenges the long-held assumption that PSA levels reliably reflect disease burden and progression, prompting a critical reevaluation of our understanding of PCa biology and disease monitoring strategies [4,5]. Indeed, the recognition of radiographic disease progression in the absence of PSA elevation raises fundamental questions about the underlying mechanisms driving PCa progression and the limitations of certain diagnostic tools in capturing the full spectrum of disease activity [9]. The clinical implications of this phenomenon are profound; it underscores the importance of incorporating imaging modalities, such as magnetic resonance imaging (MRI) and positron emission tomography (PET), into routine disease monitoring protocols, particularly in cases where PSA levels may not accurately reflect disease status [5,6,9]. It highlights the need for a more comprehensive approach to disease assessment, one that considers not only PSA kinetics but also radiographic evidence of disease progression.

Furthermore, the recognition of radiographic disease progression in PCa patients with undetectable PSA levels has significant implications for treatment decision-making. It challenges the notion that low or undetectable PSA levels necessarily correspond to favorable disease outcomes and may prompt physicians to reconsider treatment strategies for these patients [4]. Moreover, it underscores the importance of timely intervention and close monitoring in patients deemed to be at high risk of disease progression, even in the absence of PSA elevation [5]. The aim of our study is to compile the incidence rate of this phenomenon from our prospectively maintained PSMA PET registry, and to delineate the clinical ramifications for patients experiencing radiographic progression as identified through PSMA PET imaging at undetectable levels of prostate-specific antigen (referred to as “PSA zero”).

## 2. Materials and Methods

### 2.1. Patient Cohort

After obtaining approval number (22-010856) from the Mayo Clinic Institutional Review Board, we conducted a retrospective investigation using data sourced from the Mayo Clinic PSMA PET registry. The registry prospectively recorded imaging and clinical information for patients undergoing PSMA PET/CT scans between 2021 and 2023. A total of 2141 patients were included. Patients enrolled in the PSMA PET registry underwent regular physical examinations, laboratory tests (including PSA), and serial imaging in adherence to institutional guidelines to facilitate ongoing monitoring of their disease. Clinical and pathological variables were gathered, encompassing age and PSA at the time of initial PCa diagnosis, Gleason score, primary treatment for PCa, tumor staging, histological pathological subtype, prostate-specific antigen (PSA) levels, age at the time of PSA zero rDP, treatments prior to PSA zero rDP, disease status prior rDP, site of rDP at PSA zero, and subsequent clinical follow-up information. The variables were used to describe the cohort and were evaluated for any association with overall survival.

### 2.2. PSMA PET/CT Imaging

PSMA PET/CT was performed approximately every 3–6 months whenever feasible. The indication of PSMA PET/CT was based on clinical factors, including concerning findings on conventional imaging (CT, MRI, or bone scan) if performed, new or worsening symptoms such as bone pain or neurological deficit, monitoring treatment response during systemic therapy, or as part of scheduled follow-up according to the registry and institutional protocol. Imaging decisions were therefore not based solely on PSA levels, allowing detection of radiographic disease progression even in patients with PSA zero. Initial interpretations of the PSMA PET images were provided by trained nuclear medicine radiologists. Any lesions demonstrating PSMA avidity were meticulously documented. Additionally, for each lesion, the blood-pool-corrected maximum standardized uptake value (SUVmax) was measured and compared between pre- and post-treatment scans to assess interval changes following therapy. Radiographic progression was determined on PSMA PET/CT scan based on interval changes in lesion size and tracer uptake (SUVmax) over time, as well as development of new PSMA-avid lesions. Equivocal or indeterminate PET lesions were interpreted in conjunction with any available conventional imaging (MRI, CT, bone scan) to assess correlation. When such imaging was unavailable or insufficient, additional imaging including conventional modalities or C-11 Choline PET/CT was obtained as clinically appropriate. Confirmation of disease progression on PSMA PET was established by tissue histopathology whenever feasible, or alternatively by conventional imaging or subsequent PET scans.

### 2.3. Study Objectives

The primary objective was to identify patients diagnosed with prostate cancer who exhibited radiographic disease progression (rDP) on PSMA PET scans despite presenting with Zero PSA and to describe their clinicopathological variables.

The secondary objective: to report the overall survival rate among those groups of patients, which is measured from the rDP at PSA zero until death or the last date of follow-up, whichever came first.

### 2.4. Statistical Analysis

Patients were categorized into two groups based on their castrate status: hormone-sensitive prostate cancer (HSPC) and castration-resistant prostate cancer (CRPC). Descriptive statistics were employed to provide a comprehensive overview of both categorical and continuous variables, with categorical variables (including Gleason score, histopathology, tumor stage, initial treatments, treatments prior to PSA zero rDP, disease status immediately prior to PSA zero rDP) reported as frequency (percentages) and continuous variables (including age at initial diagnosis of prostate cancer, age at PSA zero rDP, PSA at initial diagnosis, time from PCa diagnosis to PSA zero rDP) reported as medians (IQR) or mean (SD). Normality of the data was assessed using the Shapiro–Wilk test. To compare nominal and continuous variables, chi-square and Kruskal–Wallis tests were utilized, respectively. Survival outcomes were assessed through overall survival (OS) using Cox regression analysis, with hazard ratios (HR) and 95% confidence intervals (CI) calculated accordingly. The Kaplan–Meier method was applied to estimate OS probability after diagnosis of PSA zero rDP according to the site of rDP, and differences in survival were evaluated using the log-rank test. All statistical tests were two-sided, and a significance level of *p* ≤ 0.05 was considered statistically significant. Statistical analyses were conducted using SPSS v28 (SPSS Inc., IBM Corp., Armonk, NY, USA).

## 3. Results

### 3.1. Baseline Characteristics Prior to PSA Zero rDP

Among the registry cohort, 257 of these men (12%) experienced rDP despite having undetectable PSA levels. Baseline clinicopathologic characteristics for these men are detailed in Table 1. At initial PCa diagnosis, the median primary Gleason score was 8 (interquartile range [IQR]: 7–9) and the median PSA level was 11.1 ng/mL (IQR: 5.6–32.7). Most of these men (61%, *n* = 157) had recurrent disease following primary treatment with radical prostatectomy or radiotherapy, while the remaining 39% (*n* = 100) presented with de novo metastatic disease. All patients had tumors available for pathologic evaluation at diagnosis, with the vast majority (97%, *n* = 250) being diagnosed with adenocarcinoma, while a small portion (3%, *n* = 7) had small cell/neuroendocrine prostate cancer evident on their earliest biopsy.

### 3.2. Clinical Features at Time of PSA Zero rDP

Over a median time of 119 months (IQR: 30.8–368.5) between the initial diagnosis of prostate cancer and the onset of PSA zero rDP, approximately 70% of patients (*n* = 184) progressed to castration-resistant prostate cancer (CRPC). Prior to the emergence of PSA zero rDP, a substantial majority (95%, *n* = 245) of patients had already developed distant metastases. Regarding treatment, 95% of these patients received at least one form of systemic therapy. Specifically, androgen deprivation therapy (ADT) was administered to 239 patients (93%), androgen receptor pathway inhibitors were used in 196 patients (77%), and chemotherapy was given to 172 patients (67%).

The patterns of disease progression at the time of PSA zero rDP are detailed in Table 2. The most frequently observed progression was bone metastasis, with or without concurrent lymph node involvement, occurring in 57% of cases. In contrast, progression involving visceral organs was relatively rare, noted in only 15% of patients (*n* = 39). Additionally, at the time of PSA zero rDP, confirmative biopsies were obtained from 18 patients (7%). Of these, 17 patients (94%) were found to have adenocarcinoma, while 1 patient (6%) exhibited small cell/neuroendocrine features.

### 3.3. Oncological Outcomes After PSA Zero rDP

**Figure 1:**
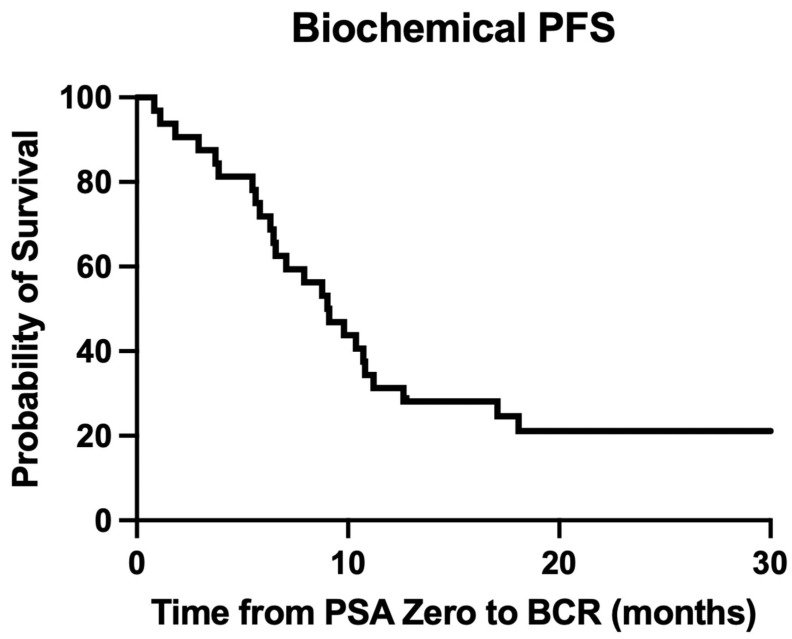
Biochemical progression-free survival (PFS) curve.

Over a median follow-up period of 8.1 months (IQR: 3.5–11.9) from the diagnosis of PSA zero rDP, 31 patients (12%) experienced biochemical recurrence, with a median biochemical progression-free survival of 9.1 months (Figure 1). Additionally, only 5% of the patients (*n* = 13) died during this follow-up period. Table 3 reveals univariate Cox regression analysis including age, Gleason score, prior treatments, CRPC status and rDP location (Local, Nodal, Bone, Visceral). Among these, the presence of visceral metastases (*p*< 0.001) was the sole factor significantly associated with worse overall survival in a univariate Cox regression analysis (HR 8.8, 95% CI 2.86–26.67, *p* < 0.0001); other variables did not reach statistical significance. Given the limited number of mortalities events (*n* = 13) and short follow-up duration, multivariate Cox regression analysis was not performed to avoid overfitting or instability of the model.

**Figure 2:**
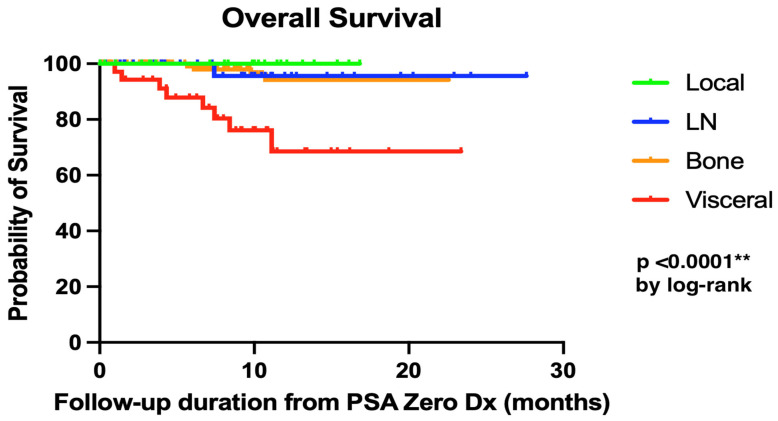
Kaplan–Meier curve (overall survival) by site of rDP. ** *p*-value < 0.01.

Furthermore, when comparing different sites of rDP, patients with visceral metastases exhibited significantly poorer overall survival (*p* < 0.0001 by log-rank test), as illustrated in Figure 2.

## 4. Discussion

Monitoring PSA levels during follow-up has historically been a highly sensitive method for the early detection of disease relapse in prostate cancer patients. Studies have shown that a deep PSA response generally correlates with improved survival outcomes [1,2,11]. Previous studies have also demonstrated that lower PSA nadir levels, deeper PSA responses, and longer time to PSA nadir after androgen deprivation therapy are associated with improved overall survival in metastatic hormone-sensitive prostate cancer [12,13]. However, recent studies have revealed significant limitations in using PSA alone to evaluate treatment response, particularly in patients with metastatic prostate cancer. For these patients, periodic imaging is essential to accurately assess disease progression [4,5]. To the best of our knowledge, our study is the largest to investigate the radiographic disease progression (rDP) in prostate cancer patients with undetectable PSA levels.

In our cohort, radiographic progression occurred despite undetectable PSA levels, suggesting that PSA alone may not fully reflect the biological activity of the disease in certain patients. Survival outcomes, particularly among those with visceral metastases, were more closely associated with imaging findings than with PSA values. These observations indicate that PSMA PET/CT can reveal clinically relevant progression even when PSA remains suppressed, especially in tumors with low or absent PSA secretion. Nevertheless, PSA remains an established prognostic marker in many clinical settings, and our findings support the combined use of PSA kinetics and advanced imaging rather than reliance on a single parameter. We analyzed data from the PSMA PET registry and identified that 12% (*n* = 257) of patients experienced PSA zero rDP, a finding that aligns closely with a previous study [5]. Notably, over 60% of this cohort had a high initial Gleason score (≥8), nearly 40% (*n* = 100) presented with de novo metastatic disease, and 72% (*n* = 184) had developed castrate-resistant disease by the time of PSA zero rDP. Furthermore, this patient group had undergone extensive treatment, with 60% receiving more than three lines of therapy. Moreover, survival outcome varied by the site of rDP. Patients with visceral metastases showed the poorest survival, with a hazard ratio of 8.8 (95% CI 2.86–26.67, *p* < 0.0001), indicating that an approximately ninefold higher risk of death compared with patients without visceral metastases involvement, while other variables including age, Gleason, prior treatment and castrate status before rDP were not statistically significant. These observations underscore the aggressive nature of the disease in this subset of patients and suggest a deviation of prostate cancer cells toward a non-PSA secretory phenotype. This shift complicates the use of PSA as a reliable biomarker and highlights the necessity for comprehensive diagnostic approaches, including advanced imaging techniques, to accurately monitor and manage disease progression in these patients.

These findings align with emerging studies that demonstrated that radiographic disease progression occurs without concordance of PSA progression [4,10,14,15,16]. A post hoc analysis of the ARCHES trial demonstrated that radiographic disease progression on conventional imaging occurred at a low level of PSA, as there was a discordance between radiographic progression and PSA progression [14].

Furthermore, multiple studies reported that PSMA PET/CT can detect prostate cancer disease recurrence, especially with low PSA levels. In prospective evaluation of PSMA PET/CT scan, Eiber et al. reported meaningful detection rates of disease recurrence at low PSA with higher rates at increasing PSA levels. Among patients with a PSA value of [0.2 < PSA < 0.5], 57.9% had positive scans, while detection rate increased with higher PSA, reaching 93% with PSA 1 to <2 ng/mL [17]. On the other hand, Treglia et al. concluded in their meta-analysis that PSMA PET/CT demonstrated higher sensitivity for detection of disease recurrence at low PSA levels. Interestingly, the sensitivity of PSMA PET/CT diminished as PSA increased [18].

Although the majority of our patients had castration-resistant prostate cancer (CRPC), not all experienced poor outcomes. Even after more than two years of follow-up from the onset of PSA zero radiographic disease progression (rDP), the median overall survival was not reached. This variability in outcomes could be attributed to the inclusion of patients with hormone-sensitive prostate cancer. Additionally, 60% of the patients in our study had metachronous prostate cancer rather than de novo metastatic disease, contributing to the observed differences in prognosis. The presence of visceral metastases, as anticipated, was associated with poorer survival outcomes, consistent with findings from previous studies [5,19,20,21]. Specifically, the study by Pezaro et al. reported that 49% of CRPC patients who developed visceral metastases did so without concurrent PSA progression, with a median survival of seven months [19]. Early detection of visceral disease is essential as it allows for biopsies and repeat molecular studies, providing critical information to inform subsequent therapeutic strategies.

Molecular studies or advanced imaging techniques such as PSMA PET/CT allow for the detection and characterization of prostate cancer based on tumor-specific biological features rather than solely on anatomical changes. Unlike the conventional imaging modalities that rely on size criteria or structure abnormalities. PSMA PET uses radiolabeled ligands that bind to the prostate-specific membrane (PSMA), which is overexpressed in most prostate cancer cells. This allows visualization of the tumor based on receptor expression and cellular activity, even when lesions are small or without structural abnormalities [22,23,24].

In our clinical practice, we advocate for the implementation of periodic advanced imaging for surveillance or evaluating treatment response in patients receiving ongoing systemic therapy [16], allowing for early detection of new or progressive disease even when PSA remained low, which could guide timely management decision.

This recommendation is supported by several potential advantages. Firstly, molecular imaging offers increased sensitivity and specificity compared to conventional imaging technologies [25,26], often eliminating the need for more invasive procedures like biopsies or surgeries. Additionally, by offering a non-invasive alternative, molecular imaging not only enhances diagnostic accuracy but also reduces the risks and discomfort associated with more invasive diagnostic procedures. Secondly, the early detection of more extensive disease development facilitates the timely implementation of therapeutic adjustments before the onset of symptomatic or functional impairment in the patient. Prompt identification and intervention in response to progressing illness may potentially postpone or even avert the decline in quality of life often associated with uncontrolled cancer progression. Finally, the emergence of metastasis-directed therapy (MDT) as a promising treatment modality for targeted management of oligometastatic disease, as evidenced in prior studies, necessitates timely imaging [27,28,29]. Delaying such imaging evaluations could lead to the inadvertent loss of the timeframe of chance for MDT. While further research is warranted to definitively establish the impact of early radiographic disease detection on overall outcomes in patients with metastatic prostate cancer, the aforementioned rationale strongly supports the clinical utility of periodic imaging surveillance.

First, one of the key strengths of this study is that we include patients with PSA zero, allowing us to detect disease recurrence before biochemical recurrence. Second, this study used large prospectively collected registry enhancing the reliability of these findings. Third, all the patients had high standardized high-quality PSMA PET/CT, ensuring accurate detection of lesions. Fourth, these results are clinically relevant as early detection of progression may help guide timely management decisions. Finally, this study provides insight into the biology of prostate cancer, showing that disease can progress with undetectable PSA possibly due to non-secreting cells or early detection on scans.

It is crucial to acknowledge the inherent limitations associated with the retrospective nature of this study design, specifically the potential for selection bias. This bias could potentially skew the observed results and limit the generalizability of the findings to the broader prostate cancer patient population. Additionally, the study population included patients at different clinical phases of prostate cancer, which introduces biological and therapeutic variability that may influence imaging findings and outcomes. This heterogeneity should be considered when interpreting the results. Furthermore, the adoption of molecular imaging such as PET scans necessitates a critical evaluation of cost-effectiveness. While PET scans offer valuable diagnostic advantages, they are typically associated with significantly higher costs compared to conventional imaging modalities [30]. Therefore, a comprehensive cost–benefit analysis is warranted to determine whether the additional information gleaned from PET scans justifies the increased financial burden. Finally, the true impact of employing serial PET scans on treatment outcomes in prostate cancer patients remains to be definitively established.

While this study provides valuable insights, it is retrospective in nature. To definitively assess the influence of serial PET scans on treatment efficacy and patient outcomes, prospective clinical trials are necessary.

## 5. Conclusions

Our study identified that some prostate cancer patients may develop radiographic disease progression even when their PSA levels are undetectable. There is a clinical conundrum, as PSA is often used without imaging to assess progression and the potential need for imaging. This study highlights the limitations of relying on PSA alone and emphasizes the value of periodic advanced imaging such as PSMA PET to better assess the disease and guide timely treatment decisions. Further prospective studies are warranted to determine how early detection of radiographic progression impacts long-term outcomes and to optimize patient management strategies.

## Figures and Tables

**Table 1:** Clinical characteristics for the entire cohort prior to PSA zero rDP.

Features	*N* = 257
**Age** at initial PCa diagnosis (Median/IQR) years	62.6 (56.9–67.7)
**PSA** at initial PCa diagnosis (Median/IQR) ng/mL	11.1 (5.6–32.7)
**Gleason score,** (Median/IQR)	8 (7–9)
**Tumor Staging**	
- Tx	145 (56%)
- T1	4 (2%)
- T2	33 (13%)
- T3	71 (28%)
- T4	4 (2%)
**Histopathology**	
- Adenocarcinoma	250 (97%)
- Neuroendocrine	7 (3%)
**Initial staging**	
- Clinically localized or regional (N1)	157 (61%)
- De novo metastatic	100 (39%)
**Initial treatment**	
- Radical prostatectomy	108 (42%)
- Radiotherapy +/− ADT	49 (19%)
- Systemic therapy alone	100 (39%)
**Time** from PCa diagnosis to PSA zero rDP, (Median/IQR) months	119 (30.8–368.5)
**Treatments prior to PSA zero rDP**	
- ADT	239 (93%)
- ARPI	196 (77%)
- Chemotherapy	172 (67%)
- Lu-177	20 (8%)
- Radium-223	1 (1%)
- PARPi	7 (3%)
- Immunotherapy	5 (2%)
- Salvage RT	111 (43%)
- Metastasis directed RT	87 (34%)
- Other	65 (25%)
**Disease status immediately prior PSA zero rDP**	
- nmHSPC	5 (2%)
- nmCRPC	7 (3%)
- mHSPC	68 (26%)
- mCRPC	177 (69%)

**Abbreviations:** PCa: prostate cancer, IQR: interquartile range, PSA: prostate-specific antigen, rDP: radiographic disease progression, NA: not available, ADT: androgen deprivation therapy, PET: positron emission tomography, ARPI: androgen receptor pathway inhibitor, Lu: lutetium, PARPi: poly (ADP-ribose) polymerase inhibitor, RT: radiotherapy, nm: non-metastatic, m: metastatic, HSPC: hormone-sensitive prostate cancer, CRPC: castrate-resistant prostate cancer.

**Table 2:** Patients’ characteristics according to the castrate-disease status.

Features	HSPC (*n* = 73)	CRPC (*n* = 184)	*p*-Value
**Age** at initial PCa diagnosis	63.4	62.5	0.445
(Median/IQR) years	(56.4–68.9)	(57.3–66.9)	
**PSA** at initial PCa diagnosis	7.9	13.9	0.074
(Median/IQR) ng/mL	(5.2–14.7)	(5.9–51.9)	
**Gleason score**			0.010 *
- Low	2 (3%)	6 (3%)	
- Intermediate	34 (47%)	50 (27%)	
- High	37 (50%)	128 (70%)	
**Tumor Staging**			<0.001 *
- Tx	27 (37%)	118 (64%)	
- ≤T2	19 (26%)	18 (10%)	
- ≥T3	27 (37%)	48 (26%)	
**Initial staging**			0.015 *
- Clinically localized or regional (N1)	58 (79%)	99 (54%)	
- De novo metastatic	15 (21%)	85 (46%)	
**Initial treatment**			<0.001 *
- Radical prostatectomy	47 (64%)	61 (33%)	
- Radiotherapy +/− ADT	11 (15%)	38 (21%)	
- Systemic therapy alone	15 (21%)	85 (46%)	
**Age** at time of PSA zero rDP	69.7	68.9	0.834
(Median/IQR) years	(64.8–75.1)	(64.4–74.9)	
**Time** from PCa diagnosis to PSA zero rDP (Median/IQR) months	49.8 (12.9–144.4)	51.9 (18.4–115.5)	0.180
**Sites of rDP**			0.039 *
- Local only	9 (12%)	17 (9%)	
- Lymph node only	17 (23%)	28 (15%)	
- Bone +/− Lymph node	38 (52%)	109 (59%)	
- Visceral +/− other	9 (12%)	30 (16%)	
**Death**			0.118
- Yes	1 (1%)	12 (7%)	
- No	72 (99%)	172 (93%)	

**Abbreviations:** HSPC: hormone-sensitive prostate cancer, CRPC: castrate-resistant prostate cancer, PCa: prostate cancer, IQR: interquartile range, PSA: prostate-specific antigen, rDP: radiographic disease progression. * *p*-value < 0.05.

**Table 3:** Univariate Cox regression analysis of factors associated with overall survival.

Features	Univariate Cox Regression	
	Hazard Ratio (95% CI)	*p*-Value
**Age** at PSA zero	0.9 (0.89–1.01)	0.123
**Gleason Score**	1.9 (0.97–3.53)	0.063
**Prior Treatment**		
ADT	1.2 (0.16–5.53)	0.838
ARPI	3.6 (0.67–18.9)	0.135
Chemo	6.2 (0.80–47.62)	0.080
**CRPC Status**	3.8 (0.49–29.62)	0.196
**rDP Location**		
Local	0.6 (0.19–2.05)	0.444
LN	1.9 (0.61–5.69)	0.277
Bone	6.3 (0.82–48.54)	0.077
Visceral	8.8 (2.86–26.67)	<0.001 *

**Abbreviation**: ADT: androgen deprivation therapy, ARPI: androgen receptor pathway inhibitor, CRPC: castrate-resistant prostate cancer, rDP: radiographic disease progression, LN: lymph node. * *p*-value < 0.05.

## Data Availability

The dataset analyzed during this study contains confidential information, and therefore restricted data can be made available upon reasonable request with permission for the purposes of peer review.

## References

[1] Matsubara N., Chi K.N., Özgüroğlu M., Rodriguez-Antolin A., Feyerabend S., Fein L., Alekseev B.Y., Sulur G., Protheroe A., Li S. (2020). Correlation of Prostate-specific Antigen Kinetics with Overall Survival and Radiological Progression-free Survival in Metastatic Castration-sensitive Prostate Cancer Treated with Abiraterone Acetate plus Prednisone or Placebos Added to Androgen Deprivation Therapy: Post Hoc Analysis of Phase 3 LATITUDE Study. Eur. Urol..

[2] Choueiri T.K., Xie W., D’Amico A.V., Ross R.W., Hu J.C., Pomerantz M., Regan M.M., Taplin M.E., Kantoff P.W., Sartor O. (2009). Time to prostate-specific antigen nadir independently predicts overall survival in patients who have metastatic hormone-sensitive prostate cancer treated with androgen-deprivation therapy. Cancer.

[3] Min K., Lin Q., Qiu D. (2025). Precision medicine in prostate cancer: Individualized treatment through radiomics, genomics, and biomarkers. Cancer Imaging.

[4] Bryce A.H., Alumkal J.J., Armstrong A., Higano C.S., Iversen P., Sternberg C.N., Rathkopf D., Loriot Y., de Bono J., Tombal B. (2017). Radiographic progression with nonrising PSA in metastatic castration-resistant prostate cancer: Post hoc analysis of PREVAIL. Prostate Cancer Prostatic Dis..

[5] Mahmoud A.M., Ahmed M.E., Kendi A.T., Thorpe M., Johnson G.B., Riaz I.B., Orme J.J., Kwon E.D., Andrews J.R., Childs D.S. (2024). Low PSA radiographic disease progression on C11-choline PET. BJUI Compass.

[6] Falchook A.D., Martin N.E., Basak R., Smith A.B., Milowsky M.I., Chen R.C. (2016). Stage at presentation and survival outcomes of patients with Gleason 8–10 prostate cancer and low prostate-specific antigen. Urol. Oncol..

[7] Valle J., von Boguslawsky K., Stenborg M., Andersson L.C. (1996). Progression from adenocarcinoma to small cell carcinoma of the prostate with normalization of prostate-specific antigen (PSA) levels. Scand. J. Urol. Nephrol..

[8] Garg I., Nathan M.A., Packard A.T., Kwon E.D., Larson N.B., Lowe V., Davis B.J., Haloi R., Mahon M.L., Goenka A.H. (2021). (11)C-choline positron emission tomography/computed tomography for detection of disease relapse in patients with history of biochemically recurrent prostate cancer and prostate-specific antigen ≤0.1 ng/mL. J. Cancer Res. Ther..

[9] Fankhauser C.D., Parry M.G., Ali A., Cowling T.E., Nossiter J., Sujenthiran A., Berry B., Morris M., Aggarwal A., Payne H. (2023). A low prostate specific antigen predicts a worse outcome in high but not in low/intermediate-grade prostate cancer. Eur. J. Cancer.

[10] Alamiri J., Britton C.J., Ahmed M.E., Andrews J.R., Higa J.L., Dundar A., Karnes R.J., Kwon E., Lowe V.J., Kendi A.T. (2022). Radiographic paradoxical response in metastatic castrate-resistant prostate cancer (mCRPC) managed with new generation anti-androgens: A retrospective analysis. Prostate.

[11] Freedland S.J., Humphreys E.B., Mangold L.A., Eisenberger M., Dorey F.J., Walsh P.C., Partin A.W. (2005). Risk of prostate cancer-specific mortality following biochemical recurrence after radical prostatectomy. JAMA.

[12] Wenzel M., Hoeh B., Hurst F., Koll F., Cano Garcia C., Humke C., Steuber T., Tilki D., Traumann M., Banek S. (2024). Impact of PSA nadir, PSA response and time to PSA nadir on overall survival in real-world setting of metastatic hormone-sensitive prostate cancer patients. Prostate.

[13] Tripathi A., Chen Y., Jarrard D.F., Garcia J.A., Dreicer R., Liu G., Hussain M.H., Shevrin D.H., Cooney M., Eisenberger M.A. (2025). Ten-year survival rates by PSA nadir in patients with metastatic hormone-sensitive prostate cancer: Long-term survival analysis from the ECOG-ACRIN 3805 (CHAARTED) trial. Ann. Oncol..

[14] Alcaraz A., Alekseev B., Shore N.D., Stenzl A., Holzbeierlein J., Zohren F., Lee H.-J., Gomez-Veiga F., Azad A., Villers A. (2022). Radiographic progression in the absence of prostate-specific antigen (PSA) progression in patients with metastatic hormone-sensitive prostate cancer (mHSPC): Post hoc analysis of ARCHES. J. Clin. Oncol..

[15] Fukuokaya W., Yanagisawa T., Mori K., Urabe F., Rajwa P., Briganti A., Shariat S.F., Kimura T. (2025). Radiographic Progression Without Corresponding Prostate-specific Antigen Progression in Patients with Metastatic Castration-sensitive Prostate Cancer Receiving Apalutamide: Secondary Analysis of the TITAN Trial. Eur. Urol. Oncol..

[16] Kleiburg F., de Geus-Oei L.F., Luelmo S.A.C., Spijkerman R., Goeman J.J., Toonen F.A.J., Smit F., van der Hulle T., Heijmen L. (2024). PSMA PET/CT for treatment response evaluation at predefined time points is superior to PSA response for predicting survival in metastatic castration-resistant prostate cancer patients. Eur. J. Radiol..

[17] Eiber M., Maurer T., Souvatzoglou M., Beer A.J., Ruffani A., Haller B., Graner F.P., Kübler H., Haberkorn U., Eisenhut M. (2015). Evaluation of Hybrid ^68^Ga-PSMA Ligand PET/CT in 248 Patients with Biochemical Recurrence After Radical Prostatectomy. J. Nucl. Med..

[18] Treglia G., Pereira Mestre R., Ferrari M., Bosetti D.G., Pascale M., Oikonomou E., De Dosso S., Jermini F., Prior J.O., Roggero E. (2019). Radiolabelled choline versus PSMA PET/CT in prostate cancer restaging: A meta-analysis. Am. J. Nucl. Med. Mol. Imaging.

[19] Pezaro C., Omlin A., Lorente D., Rodrigues D.N., Ferraldeschi R., Bianchini D., Mukherji D., Riisnaes R., Altavilla A., Crespo M. (2014). Visceral disease in castration-resistant prostate cancer. Eur. Urol..

[20] Satapathy S., Mittal B.R., Sood A. (2020). Visceral Metastases as Predictors of Response and Survival Outcomes in Patients of Castration-Resistant Prostate Cancer Treated With 177Lu-Labeled Prostate-Specific Membrane Antigen Radioligand Therapy: A Systematic Review and Meta-analysis. Clin. Nucl. Med..

[21] Yanagisawa T., Rajwa P., Kawada T., Mori K., Fukuokaya W., Petrov P., Quhal F., Laukhtina E., von Deimling M., Bianchi A. (2023). Efficacy of Systemic Treatment in Prostate Cancer Patients With Visceral Metastasis: A Systematic Review, Meta-analysis, and Network Meta-analysis. J. Urol..

[22] Shen Z., Li Z., Li Y., Tang X., Lu J., Chen L., Cheng Z.Z., Liao H., Zhou S. (2025). PSMA PET/CT for prostate cancer diagnosis: Current applications and future directions. J. Cancer Res. Clin. Oncol..

[23] Ceci F., Castellucci P., Fanti S. (2019). Current application and future perspectives of prostate specific membrane antigen PET imaging in prostate cancer. Q. J. Nucl. Med. Mol. Imaging.

[24] Seifert R., Gafita A., Telli T., Voter A., Herrmann K., Pomper M., Hadaschik B., Rowe S.P., Fendler W.P. (2024). Standardized PSMA-PET Imaging of Advanced Prostate Cancer. Semin. Nucl. Med..

[25] Farolfi A., Calderoni L., Mattana F., Mei R., Telo S., Fanti S., Castellucci P. (2021). Current and Emerging Clinical Applications of PSMA PET Diagnostic Imaging for Prostate Cancer. J. Nucl. Med..

[26] Zhang H., Xie C., Huang C., Jiang Z., Tang Q. (2025). Comparison of Prostate-Specific Membrane Antigen Positron Emission Tomography and Conventional Imaging Modalities in the Detection of Biochemical Recurrence of Prostate Cancer and Assessment of the Role of Artificial Intelligence: A Systematic Review and Meta-analysis. Acad. Radiol..

[27] Decaestecker K., De Meerleer G., Ameye F., Fonteyne V., Lambert B., Joniau S., Delrue L., Billiet I., Duthoy W., Junius S. (2014). Surveillance or metastasis-directed Therapy for OligoMetastatic Prostate cancer recurrence (STOMP): Study protocol for a randomized phase II trial. BMC Cancer.

[28] Phillips R., Shi W.Y., Deek M., Radwan N., Lim S.J., Antonarakis E.S., Rowe S.P., Ross A.E., Gorin M.A., Deville C. (2020). Outcomes of Observation vs. Stereotactic Ablative Radiation for Oligometastatic Prostate Cancer: The ORIOLE Phase 2 Randomized Clinical Trial. JAMA Oncol..

[29] Chen W.S., Tuchayi A.M., Sabbagh A., Kim I., Porter E., Ashraf-Ganjouei A., Li Y.R., Witztum A., Rajagopal A., Seyedin S.N. (2025). Utility of metastasis-directed radiotherapy with and without hormonal therapy in management of oligometastatic prostate cancer. JNCI Cancer Spectr..

[30] Boustani A.M., Pucar D., Saperstein L. (2018). Molecular imaging of prostate cancer. Br. J. Radiol..

